# YouTube: Is It a Reliable Source of Nutrition Information on COVID-19 Pandemic?

**DOI:** 10.3390/healthcare10101911

**Published:** 2022-09-29

**Authors:** Elif Inan-Eroglu, Zehra Buyuktuncer

**Affiliations:** 1 Charles Perkins Centre, School of Health Sciences, Faculty of Medicine and Health, The University of Sydney, Sydney, NSW 2050, Australia; 2 Department of Nutrition and Dietetics, Faculty of Health Sciences, Hacettepe University, Ankara 06100, Turkey

**Keywords:** YouTube, 2019 novel corona virus, COVID-19, nutrition, misinformation

## Abstract

Data on the nutrition-related misinformation about COVID-19 are limited. This study analysed the quality and accuracy of the nutrition information available on YouTube about current COVID-19 pandemic as well as assessed the content of the videos. YouTube was searched using the terms “nutrition and COVID-19” in Turkish on 1 February 2021. Videos were filtered according to relevancy, and the first 280 videos were analysed. A total of 218 videos were reviewed and classified as “misleading” or “relevant” depending on the information provided. The transparency, utility, reliability, and accuracy of video contents were assessed. The videos attracted a cumulative 6,258,694 views. There were 178 (81.7%) fully relevant and 40 (18.3%) misleading videos. Approximately 80% of the videos shared by health professionals were relevant videos. Government organisations only shared relevant videos. Relevant videos had higher reliability, accuracy, and quality than misleading videos. The nutrition-related content of COVID-19 videos is suboptimal on YouTube. As the COVID-19 pandemic worsens, and nutrition could improve immunity, health professionals and educational and government organisations need to engage more in the spread of nutrition-related COVID-19 information to Internet platforms based on nutrition guidelines and the latest scientific evidence. This will be a practical and immediately implementable public health strategy to effectively spread the right information.

## 1. Introduction

Coronavirus disease 2019 (COVID-19), caused by the severe acute respiratory syndrome coronavirus 2 (SARS-CoV-2), was first reported in Wuhan City of China in December 2019. The virus has rapidly spread to every inhabited continent worldwide and thus became a major public health emergency of international concern [1]. In March 2020, the World Health Organization (WHO) declared COVID-19 as a global pandemic [2]. As of 21 September 2022, more than 618 million were infected with the COVID-19 disease, and more than 6 million deaths have been reported globally [3].

The Internet is the easiest way to obtain quick and broad information about health for the public in the current digital world [4,5]. A popular open-access video hosting website, YouTube (www.youtube.com), with over two billion users and an estimated monthly viewership of one billion, is one of the most prevalent sources of health-related online information [6]. However, the lack of an editorial peer-review process and the diversity of authorship of origin on YouTube may allow the spread of misleading or inaccurate health-related information that is not supported by scientific evidence [7,8,9]. The quality and the accuracy of COVID-19-related information on YouTube has been evaluated previously [7,10,11]. YouTube viewership during the COVID-19 outbreak is higher than previous outbreaks [10], including the Ebola outbreak in 2014 [12] and the Zika virus epidemic in 2016 [13], and over one-quarter of the most-viewed YouTube videos on COVID-19 contained misleading information, indicating that the medical content of videos is suboptimal [7]. Additionally, the international health agencies are underrepresented in YouTube [10], and misleading videos had more likes, fewer comments, and longer running times than useful videos [11].

Although studies about the COVID-19-related information on YouTube provide preliminary insights into the accuracy and quality of the videos, these studies did not consider nutrition-related COVID 19 information. Since one of the most searched topics about health on the Internet is nutrition, and to our knowledge, there is no international consensus about nutrition during the COVID-19 pandemic, it is crucial to give the best scientific nutrition-related information to the public. The aim of this study is to analyse the quality and accuracy of the nutrition information available on YouTube about the current COVID-19 pandemic as well as to assess the content of the videos.

## 2. Materials and Methods

The online video hosting resource YouTube (http://www.youtube.com) was accessed on 1 February 2021 from Turkey using the search term “Coronavirus and nutrition” in Turkish language. The use of additional search terms was deemed as unnecessary, as preliminary searches illustrated large content overlap between different search terms, for example, “COVID-19 and nutrition”. The search was done using a cleared-cache web browser, which consists of the most recent version of Google Chrome in incognito mode with all available updates installed. The first 280 videos from search were included for further analysis. This screening strategy is based on previous studies indicating that the users do not go beyond the first few pages of results from a search engine [14,15,16]. The results were sorted in decreasing order of relevance using the default YouTube algorithm. These 280 videos were saved in a playlist for further analysis because the search results in YouTube can change on a day-to-day basis [12]. Uniform resource locators (URLs) of these videos were also saved separately as back up. Our search methodology is in alignment with previous studies on YouTube content [17,18].

Exclusion criteria were as follows: videos in language other than Turkish, absence of audio and/or visual information, videos lacking information on the 2019 novel coronavirus and nutrition, duplicate videos, and livestream videos. As this study required analysis of publicly available information, Institution Review Board approval was not required.

### 2.1. Data Extraction

The preliminary review of the videos was performed by nine intern dietitians. Subsequently, all videos were independently reviewed and analysed by two reviewers (E.I.E. and Z.B.) who are experts in the field of nutrition and dietetics, and the results were then consolidated. Any discrepancies were resolved by an external reviewer who is also expert in nutrition and dietetics. Videos were classified as “relevant” if they contained scientifically correct information about nutrition during COVID 19 and as “misleading” if they contained scientifically unproven information. Videos were considered misleading if they contained one or more misleading scientifically unproven statements, as evaluated based on guidelines from major public health agencies (e.g., World Health Organization [19,20,21,22], Turkish Dietetic Association [23,24], and National Institute for Health and Care Excellence of England [25]) at the video’s publication date. We considered a video misleading if it contained any misleading information in addition to scientifically proven information because it still had the potential to disseminate misinformation.

Descriptive characteristics of all videos, including video title, video hyperlink, number of views, number of likes and dislikes, the like ratio (like × 100/[like + dislike]), upload date of video, video duration, the view ratio (number of views/days), number of comments, and video source category were included. The video power index (VPI) was also calculated by the following formula: like ratio × view ratio/100 to evaluate the popularity of the videos [26]. The upload source of each video was identified and categorized into 7 groups, namely news channels, health professionals (including doctors, dietitians, and nurses), health centres, TV channels, government organisations, educational organisations, and independent individual channels, based on the information given at “about” section of their YouTube profile. The definition of the upload sources is given in the Table S1.

### 2.2. Categorization of Video Content

All videos were analysed for reliability, quality (in term of content and audio-visual characteristics), title–content consistency, and accuracy. The full list of the scoring systems is given in the Tables S2–S8.

The transparency, utility, reliability, and accuracy of video content was assessed using the Journal of the American Medical Association (JAMA) benchmark criteria and modified DISCERN score (mDISCERN) [16,17,27]. By assigning 1 point for the presence of each criterion, the total JAMA score (JAMAS) was calculated (range from 0 to 4; 1 point—insufficient data about video source, 2–3—partially sufficient data about video source, 4—completely sufficient data about video source). The mDISCERN score assesses clarity, reliability, bias, reference supplementation, and areas of uncertainty for information in YouTube videos. One point is awarded for each criterion, and the maximum total score is 5 points, which indicates the highest reliability. In addition to JAMAS and modified DISCERN score, the accuracy of video content was determined using accuracy score (AS) (0: misleading and grossly inaccurate; 1—poor, easily identified inaccurate information; 2—average, some oversimplification, overall correct information; 3—excellent, professional level, highly accurate) [28]. Comprehensiveness score (CS) was used to assessed whether the videos cover the relevant information to the topic (0—no information provided; 1—poor, lacking important information; 2—average, covers most relevant information; 3—excellent, covers all relevant information) [28].

Global quality score (GQS) was assessed for using a 5-point global score the educational value of each video (1—poor quality, very unlikely to be of any use to patients; 2—poor quality but some information present, of very limited use to patients; 3—suboptimal flow, some information covered, but important topics are missing, somewhat useful to patients; 4—good quality and flow, most important topics covered, useful to patients; 5—excellent quality and flow, highly useful to patients) [16].

Audio-visual quality (AVQ) score was used to assess the auditory and visualisation quality of the videos (0—unable to view; 1—poor, blurry, out of focus, unintelligible; 2—average, non-professional editing; 3—excellent, clear, professional editing) [28].

A scoring system called title–content consistency index (TCCI) was used to assess video title and content consistency. TCCI is a 5-point Likert scale ranging from 1 (poor consistency) to 5 (high consistency), which rates the gap between title and content [11].

All videos included COVID-19- and nutrition-related information. The videos were further categorized as regarding boosting immune system, the importance of physical activity and child nutrition during the pandemic, and recommendations for people with chronic diseases.

### 2.3. Statistical Analysis

All statistical analysis was performed using SSPS software (SSPS 20.0, IBM, Chicago, IL, USA). Descriptive statistics (mean, standard deviation, frequency, percentage) were used to evaluate study data. Categorical variables were presented as frequencies and continuous variables were reported as means. Inter reviewer agreement for relevant/misleading videos was analysed using intraclass correlation coefficient (ICC) estimates. We considered ICC > 0.90 as excellent; 0.75 to <0.90 as good; 0.50 to <0.75 as moderate; and <0.50 as poor. The Shapiro–Wilk test was used to approximate normality of quantitative data. The Kruskal–Wallis test for intergroup comparisons and the Mann–Whitney U test for two-group comparison were used for non-normally distributed continuous variables. Spearman correlation was used for the assessment of the relationship between quantitative characteristics of the videos and different scoring systems as well as the correlation between different scoring systems. A *p*-value of less than 0.05 was considered significant.

## 3. Results

The characteristics of the videos are given in Table 1. The videos attracted a cumulative 6,258,694 views. The mean number of views (SD) was 28,709.6 (113,804.8). The mean like ratio (SD) was 89.8 (26.5), and the mean length of the videos (SD) was 11.2 (15.5) minutes. More than 80% of the videos were relevant to the topic, whereas around 20% of them included misleading information. There was excellent reliability between reviewers for relevant/misleading videos (ICC range = 0.90–1.00). Table S9 gives the examples of misleading information. Relevant videos (90.6) had a higher like ratio than misleading videos (86.6). Relevant videos had higher scores for all quality, reliability, accuracy, and comprehensiveness scoring systems included to the study than misleading videos.

Table 2 shows the source and content wise distribution of the included videos. Health professionals, including doctors, dietitians, and nurses, shared 30.7% videos, whereas 18.7% of the videos were shared by independent users. Educational organisations such as universities and colleges uploaded only 5% of the total videos. Approximately 80% of the videos that shared by health professionals were relevant videos. Independent users shared 25% of the misleading videos. All of the videos that were uploaded by government organisations were relevant videos. The majority of shared videos were about boosting immune system (71.6%), followed by the importance of physical activity (3.7%). Approximately 20% of the boosting immune system videos and 25% of importance of physical activity videos were misleading.

Videos shared by government organisations had the highest scores among the videos uploaded by different sources according to most of the scoring systems (like ratio (97.9), mDISCERN score (3.1), AS (2.4), GQS (3.5), CS (2.3), TCCI (3.8)) (Table 3). TV channels’ videos had a higher view ratio and AVQ (350.9 and 2.5, respectively) compared to the other sources. There was no statistically significant difference between content of the videos in terms of quality, reliability, accuracy, or comprehensiveness scoring systems (Table 3).

Table 4 presents the association between quantitative characteristics of the videos and different scoring systems. There was a positive correlation between mDISCERN score with the number of views and view ratio (r = 0.141, *p* < 0.05 and r = 0.177, *p* < 0.05, respectively), and a negative correlation between AS and the number of dislikes (r = −0.134, *p* < 0.05). Table S10 shows the correlation between different scoring systems. There was a positive association between mDISCERN score and AVQ with other scoring systems.

## 4. Discussion

Our study highlighted the importance of YouTube as an online platform for sharing of nutrition-related COVID-19 information. The wide range of channel sources and the number of shared videos demonstrated a growing interest to nutrition in COVID 19.

The analysis regarding the information given in the videos showed that approximately 20% of the videos were misleading, containing scientifically wrong or unproven nutrition-related COVID-19 information. Although this finding was in line with the previous studies that found that approximately 15–30% of the information on YouTube videos related to diseases can misinform the public [14,15], a recent study by Khatri et al. [10] on the content analysis of YouTube videos on COVID-19 found that only two videos were misleading amongst the included 72 videos. The higher percentage of misleading videos in our study can be explained by the lack of nutrition consensus during COVID-19, which consequently can lead to disinformation. In parallel with our findings, a recent study that evaluated English-language videos addressing COVID-19 showed that 27.5% of the included videos were misleading [7], whereas a Korean study found that 37.1% of COVID-19-related videos were misleading [11].

In terms of the number of views, misleading videos were viewed more than twice more than the relevant videos in this study; however, the difference was not statistically significant. Previous studies on COVID-19-,related YouTube videos also showed that the number of views for the misleading videos was higher than relevant/useful videos [7,10,11]. In contrast, relevant videos had higher reliability, accuracy and quality than misleading videos.

One-fourth of the videos that were shared by independent users were misleading. In addition, the most misleading content was about the boosting immune system. For example, “homemade yoghurt is a probiotic food”, “people should follow vegetarian diet and avoid meat and meat products in order to boost their immune system”, and “gluten and dairy products can harm the gut and therefore decrease immune response”.

Government organisations shared only relevant videos. Similarly, in a study by Li et al. [7] and Moon and Lee [11], it was also shown that although government-generated videos comprised only a small percentage of the shared videos, they contained only useful information and were effective delivery tools, which had higher reliability and overall quality.

Our study has several notable strengths, including the large number of videos (*n* = 280). We had detailed information regarding reliability, accuracy, and quality (both content and audio-visual) of the videos by using a wide range of different scoring systems. There are also several limitations to this study. First, our video search only represents the state of YouTube at a single time point. This can lead to different results if the search is conducted at a different point in time, as the viewership and content on YouTube is dynamic and therefore might change rapidly on a daily basis. Nonetheless, the search for the videos was conducted more than one year after the first confirmed case, which indicates that videos may have a standard ranking. Second, the analysed videos were limited to the Turkish language, which limits the generalizability of the findings to different languages. However, similar results were shown about COVID-19-related information across different languages [7]. Lastly, only YouTube was used for the video search, and the same videos present on other websites were not considered.

## 5. Conclusions

The nutrition-related content of COVID-19 videos is suboptimal on YouTube. With the limited regulatory oversight around the scientific quality and content of shared nutrition information online, health professionals and educational and government organisations need to be aware of video-sharing websites such as YouTube, given that misinformation on nutrition is a significant risk factor for engaging in risky health behaviours, and respond to the public, who generally obtain their nutrition information online. Our findings have the potential to inform health-promotion practices, and our study revealed that more research is warranted into nutrition information not for only COVID-19-related but in general for shared videos on YouTube. As the COVID-19 pandemic worsens, and nutrition could improve immunity, health professionals and educational and government organisations need to more engage in the spread of nutrition-related COVID-19 information to Internet platforms such as YouTube based on nutrition guidelines and the latest scientific evidence and to minimise the spread of misinformation. This will be a practical and immediately implementable public health strategy to effectively spread the right information.

## Figures and Tables

**Table 1:** Characteristics of videos included for analysis.

	Total Videos (*n* = 218)	Relevant Videos (*n* = 178)	Misleading Videos (*n* = 40)	*p*-Value *
Number of views	28,709.6 (113,804.8)	24,975.3 (103,042.1)	45,327.3 (153,391.4)	0.582
View ratio	173.3 (691.3)	154.5 (686.3)	257.0 (715.9)	0.421
Number of likes	432.0 (1699.3)	407.0 (1771.2)	543.6 (1346.7)	0.279
Number of dislikes	18.5 (80.0)	14.7 (66.5)	35.7 (123.2)	*0.040*
Like ratio	89.8 (26.5)	90.6 (25.9)	86.6 (29.5)	*0.003*
Number of advertisements	1.9 (1.2)	1.7 (1.2)	2.6 (1.1)	*0.018*
Number of comments	44.8 (170.9)	40.4 (169.6)	64.4 (177.6)	0.137
Length of videos (mins)	11.2 (15.5)	10.9 (15.6)	12.6 (15.2)	0.107
VPI	165.5 (665.6)	148.5 (666.4)	241.0 (665.4)	0.338
JAMAS	1.9 (0.7)	1.9 (0.7)	1.8 (0.7)	0.446
mDISCERN	2.5 (1.4)	2.8 (1.3)	1.2 (0.8)	*<0.001*
AS	2.0 (0.8)	2.3 (0.5)	0.7 (0.6)	*<0.001*
GQS	3.1 (1.2)	3.5 (1.1)	1.6 (0.6)	*<0.001*
CS	1.8 (0.7)	1.9 (0.6)	0.9 (0.5)	*<0.001*
TCCI	3.4 (1.1)	3.7 (1.0)	2.4 (1.3)	*<0.001*
AVQ	2.3 (0.7)	2.4 (0.6)	2.0 (0.6)	*<0.001*

Data are presented as mean (standard deviation). * Mann–Whitney U Test. VPI, video power index; JAMAS, *Journal of the American Medical Association* (JAMA) score; mDISCERN, modified DISCERN score; AS, accuracy score; GQS, global quality score; CS, comprehensiveness score; TCCI, title–content consistency index; AVQ, audio-visual quality. Italic font indicates statistical significance (*p* < 0.05).

**Table 2:** Source and content wise distribution of videos (*n* = 218).

	Total Videos	Relevant Videos	Misleading Videos
*Upload Source of the Videos*			
News channels	25 (11.5)	21 (11.8)	4 (10.0)
Health professionals	67 (30.7)	53 (29.8)	14 (35.0)
Health centres	31 (14.2)	29 (16.3)	2 (5.0)
TV channels	27 (12.4)	18 (10.1)	9 (22.5)
Government organisations	17 (7.8)	17 (9.6)	-
Educational organisations	11 (5.0)	10 (5.6)	1 (2.5)
Independent users	40 (18.3)	30 (16.9)	10 (25.0)
*Content of the Videos*			
Boosting immune system	156 (71.6)	126 (70.8)	30 (75.0)
Importance of physical activity	8 (3.7)	6 (3.4)	2 (5.0)
Child nutrition during pandemic	4 (1.8)	4 (2.2)	-
Recommendations for people with chronic diseases	7 (3.2)	6 (3.4)	1 (2.5)

Data are presented as *n* (percentage).

**Table 3:** Mean like ratio, view ratio, VPI, AVQ, accuracy score, CS, GQS, CCI, RA, and JAMAS values of the videos based on source and content.

	Like Ratio	View Ratio	VPI	JAMAS	mDISCERN	AS	GQS	CS	TCCI	AVQ
*Upload Source of the Videos*										
News channels	83.9 (32.1)	151.6 (503.5)	136.1 (449.0)	2.0 (0.6)	2.6 (1.6)	2.0 (0.7)	3.0 (1.2)	1.7 (0.7)	3.3 (1.2)	2.1 (0.7)
Health professionals	94.1 (20.9)	185.1 (638.7)	180.7 (624.4)	1.9 (0.7)	2.6 (1.3)	2.1 (0.8)	3.1 (1.3)	1.7 (0.7)	3.5 (1.1)	2.3 (0.7)
Health centres	73.3 (44.1)	3.8 (7.1)	3.5 (7.2)	2.2 (0.8)	2.6 (1.2)	2.2 (0.8)	3.4 (1.1)	1.9 (0.7)	3.6 (1.0)	2.4 (0.6)
TV channels	89.5 (26.0)	350.9 (866.0)	323.6 (797.5)	1.7 (0.7)	2.3 (1.2)	1.8 (0.9)	2.8 (1.2)	1.5 (0.8)	3.2 (1.3)	2.5 (0.6)
Government organisations	97.9 (6.1)	2.9 (3.1)	2.8 (3.0)	2.1 (0.7)	3.1 (1.1)	2.4 (0.5)	3.5 (1.1)	2.3 (0.7)	3.8 (1.2)	2.2 (0.5)
Educational organisations	84.5 (29.5)	1.5 (2.4)	1.4 (2.4)	1.4 (0.5)	2.3 (1.4)	2.0 (0.9)	3.3 (1.2)	2.0 (0.6)	3.4 (1.0)	2.3 (0.6)
Independent users	97.6 (3.7)	298.4 (1109.1)	291.4 (1087.7)	1.7 (0.7)	2.2 (1.6)	1.8 (1.0)	2.9 (1.3)	1.6 (0.7)	3.3 (1.3)	2.3 (0.6)
*p* *	0.044	0.006	0.001	0.012	0.310	0.262	0.424	0.025	0.638	0.187
*Content of the Videos*										
Boosting immune system	89.3 (27.4)	211.3 (797.0)	201.8 (767.7)	1.9 (0.7)	2.7 (1.4)	2.0 (0.8)	3.2 (1.2)	1.8 (0.7)	3.5 (1.1)	2.3 (0.6)
Importance of physical activity	85.3 (34.5)	196.5 (331.5)	187.5 (313.0)	1.9 (0.6)	1.7 (0.9)	1.4 (0.9)	2.5 (1.2)	1.2 (0.7)	2.9 (1.4)	2.2 (0.7)
Child nutrition during pandemic	95.8 (8.3)	6.0 (4.6)	6.0 (4.6)	1.7 (0.5)	2.7 (1.7)	2.2 (0.5)	3.7 (1.2)	2.0 (0.8)	4.0 (0.8)	2.2 (0.5)
Recommendations for people with chronic diseases	85.0 (37.5)	4.8 (8.1)	4.2 (8.2)	2.2 (0.5)	2.0 (1.4)	2.0 (1.0)	3.0 (1.3)	1.6 (1.0)	3.1 (1.3)	2.6 (0.5)
*p* *	0.640	0.772	0.599	0.703	0.204	0.217	0.345	0.127	0.401	0.764

Data are presented as mean (standard deviation). * Kruskal–Wallis Test. VPI, video power index; JAMAS, *Journal of the American Medical Association* (JAMA) score; mDISCERN, modified DISCERN score; AS, accuracy score; GQS, global quality score; CS, comprehensiveness score; TCCI, title–content consistency index; AVQ, audio-visual quality.

**Table 4:** Assessment of the relationship between quantitative variables and scores.

		VPI	JAMAS	mDISCERN	AS	GQS	CS	TCCI	AVQ
Length	r	0.314	0.074	0.128	0.061	0.193	0.199	0.233	0.112
	*p*	*p* < 0.01	0.304	0.070	0.370	0.004	*p* < 0.01	*p* < 0.01	0.099
View	r	0.852	0.126	0.141	0.045	0.111	0.017	0.116	0.303
	*p*	*p* < 0.01	0.079	0.045	0.506	0.101	0.807	0.087	*p* < 0.01
Like	r	0.815	0.048	0.049	0.015	0.067	−0.022	0.078	0.205
	*p*	*p* < 0.01	0.509	0.486	0.828	0.326	0.743	0.254	*p* < 0.01
Dislike	r	0.684	0.036	−0.001	−0.134	−0.040	−0.123	−0.009	0.178
	*p*	*p* < 0.01	0.616	0.989	0.048	0.562	0.069	0.895	*p* < 0.01
View Ratio	r	0.975	0.097	0.177	0.047	0.104	0.022	0.117	0.294
	*p*	*p* < 0.01	0.178	0.012	0.492	0.128	0.743	0.084	*p* < 0.01
Like ratio	r	−0.154	0.05	0.030	0.208	0.140	0.144	0.147	−0.025
	*p*	0.023	0.941	0.667	*p* < 0.01	0.039	0.034	0.0.30	0.716

r, Spearman’s rho; VPI, video power index; JAMAS, *Journal of the American Medical Association* (JAMA) score; mDISCERN, modified DISCERN score; AS, accuracy score; GQS, global quality score; CS, comprehensiveness score; TCCI, title–content consistency index; AVQ, audio-visual quality.

## Data Availability

The YouTube is an open-access resource.

## Supplementary Materials

The following supporting information can be downloaded at: https://www.mdpi.com/article/10.3390/healthcare10101911/s1, Table S1: Video publishing category descriptions, Table S2: Modified DISCERN Score, Table S3: *Journal of the American Medical Association* Score (JAMAS), Table S4: Audio-Visual Quality, Table S5: Accuracy Score, Table S6: Comprehensiveness Score, Table S7: Title–content consistency index, Table S8: Global Quality Score for educational value, Table S9: Examples of misleading nutrition information, Table S10: Determining the relationship level between scores.
