# Supplementary material for: YouTube: Is It a Reliable Source of Nutrition Information on COVID-19 Pandemic?
Source: Healthcare (Basel). 2022 Sep 29;10(10):1911. doi: 10.3390/healthcare10101911 (PMC9601637; doi:10.3390/healthcare10101911)
Supplement: Supplementary file 1 [file healthcare-10-01911-s001.zip › healthcare-1911759-Supplementary.pdf]

Supplementary Table S1: Video publishing category descriptions.

**Video Source Category**	**Description**
News channels	Created by news channels.
Health professionals	Created by doctors, dietitians, nurses, pharmacist.
Health centres	Created by hospital or inpatient services.
TV channels	Created by TV channels.
Government organisations	Uploaded by a government agency or organisation.
Educational organisations	Created by educational organisations including universities.
Independent users	Individual or group of individuals with no professional credentials or established organizational affiliation.

Supplementary Table S2. Modified DISCERN Score.

1. Are the aims clear and achieved?<br>2. Are reliable sources of information used? (i.e., publication cited, speaker is a certified physician)<br>3. Is the information presented balanced and unbiased?<br>4. Are additional sources of information listed for patient reference?<br>5. Are areas of uncertainty mentioned?

Supplementary Table S3. Journal of the American Medical Association Score (JAMAS).

Authorship	Authors and contributors, their affiliations, and relevant credentials should be provided
Attribution	References and sources for all content should be listed clearly, and all relevant copyright information should be noted
Disclosure	Website "ownership" should be prominently and fully disclosed, as should any sponsorship, advertising, underwriting, commercial funding arrangements or support, or potential conflicts of interest
Currency	Dates when content was posted and updated should be indicated

Supplementary Table S4. Audio-Visual Quality.

3 - Excellent - Clear, professional editing<br>2 - Average - non-professional editing<br>1 - Poor - blurry, out of focus, unintelligible<br>0 - Unable to view

Supplementary Table S5. Accuracy Score.

3 - Excellent - Professional level, highly accurate<br>2 - Average - Some oversimplification, overall correct information<br>1 - Poor - Easily identified inaccurate information<br>0 - Misleading and grossly inaccurate

Supplementary Table S6. Comprehensiveness Score.

3 - Excellent - Covers all relevant information<br>2 - Average - Covers most relevant information<br>1 - Poor - Lacking important information<br>0 - No information provided

Supplementary Table S7. Title–content consistency index.

Number of statements	Statements
1	Eye-catching thumbnail pictures and sensationalist headlines to capture attention, but video title does not match the content
2	Visually attractive thumbnail, strong emotionally appealing headlines, and only a little relevant information listed
3	Some relevant information listed, but large gaps between title and its content
4	Most of the relevant information discussed, but small gaps between title and its content
5	An excellent title for the content

Supplementary Table S8. Global Quality Score for educational value.

1 Poor quality; very unlikely to be of any use to patients.
2 Poor quality but some information present; of very limited use to patients.
3 Suboptimal flow, some information covered but important topics missing; somewhat useful to patients.
4 Good quality and flow, most important topics covered; useful to patients.
5 Excellent quality and flow; highly useful to patients.

Supplementary Table S9. Examples of misleading nutrition information.

Misleading Information
You should not eat foods containing gluten during pandemic.
Homemade yoghurt is probiotic food.
Intermittent fasting should be suggested during pandemic.
Pistachio can prevent from COVID-19.

Supplementary Table S10. Determining the relationship level between scores.

		VPI	JAMAS	mDISCERN	AS	GQS	CS	TCCI	AVQ
VPI	r		0.078	0.145*	0.043	0.100	0.012	0.125	0.276**
	p		0.279	0.040	0.529	0.140	0.863	0.064	p < 0.01
JAMAS	r	0.078		0.352**	0.220**	0.188**	0.237	0.242**	0.130
	p	0.279		p < 0.01	p < 0.01	p < 0.01	0.001	p < 0.01	0.071
mDISCERN	r	0.145*	0.352**		0.434**	0.545**	0.600**	0.409**	0.222**
	p	0.040	p < 0.01		p < 0.01	p < 0.01	0.00	p < 0.01	p < 0.01
AS	r	0.043	0.220**	0.434**		0.773**	0.665**	0.579**	0.311**
	p	0.529	0.002	p < 0.01		p < 0.01	0.00	p < 0.01	p < 0.01
GQS	r	0.100	0.188**	0.545**	0.773**		0.791**	0.771**	0.287**
	p	0.140	p < 0.01	p < 0.01	p < 0.01		p < 0.01	p < 0.01	0 p < 0.01
CS	r	0.012	0.237	0.600**	0.665**	0.791**		0.764**	0.236**
	p	0.863	0.001	p < 0.01	p < 0.01	p < 0.01		p < 0.01	0.001
TCCI	r	0.125	0.242**	0.409**	0.579**	0.771**	0.764**		0.234**
	p	0.064	p < 0.01	p < 0.01	p < 0.01	p < 0.01	p < 0.01		0.001
AVQ	r	0.276**	0.130	0.222**	0.311**	0.287**	0.236**	0.234**	
	p	p < 0.01	0.071	p < 0.01	p < 0.01	p < 0.01	p < 0.01	p < 0.01	

r, Spearman's rho *p < 0.05, **p < 0.01, ***p < 0.001
